# Harnessing digital technology to predict, diagnose, monitor, and develop treatments for brain disorders

**DOI:** 10.1038/s41746-019-0123-z

**Published:** 2019-06-03

**Authors:** Clare Stroud, Jukka-Pekka Onnela, Husseini Manji

**Affiliations:** 1 The National Academies of Sciences, Engineering, and Medicine, 500 Fifth Street NW, Washington, DC 20001 USA; 2 Department of Biostatistics, Harvard T.H. Chan School of Public Health, Boston, MA 02115 USA; 3 Janssen Research and Development, LLC, Raritan, NJ 08869 USA

**Keywords:** Biomarkers, Neurological disorders, Psychiatric disorders, Neurological disorders, Health policy

## Abstract

Digital technologies—including smartphones, wearables, and social media data—show great potential for helping to alleviate suffering from brain disorders such as Parkinson’s disease, Alzheimer’s disease, depression, and schizophrenia. However, as researchers, technology developers, disease-focused groups, and others forge forward to take advantage of the tremendous opportunities in this domain, it is important to avoid hype and overpromising, and to ensure that this work is done rigorously and collaboratively. In June 2018, the National Academies of Sciences, Engineering, and Medicine’s Forum on Neuroscience and Nervous System Disorders hosted a workshop that brought together a wide range of experts and stakeholders. The workshop provided an opportunity to take stock of the rapidly-evolving landscape and discuss how to work together to address both scientific and practical challenges, so that the potential of digital technologies can be translated into meaningful contributions toward the health of individuals and society. Workshop presentations and discussions focused on four key challenges: transforming data into insight, navigating regulatory pathways, designing user-centered tools, and building partnerships across a complex ecosystem. This article highlights the many issues, challenges, and opportunities discussed by individual participants at the workshop.

## Introduction

The past few years have witnessed an explosion of interest in harnessing digital technologies—including smartphones, wearables, and social media data—to help alleviate suffering from central nervous system (CNS) disorders. These disorders, such as Parkinson’s disease, Alzheimer’s disease, mood disorders, and schizophrenia, are leading causes of disability and death worldwide.^1^ Despite the tremendous need for effective treatment, development of therapies for these conditions has lagged, in large part because of our poor understanding of the underlying neurobiology; the complexity of phenotypes, behavior, and symptoms; and in most cases a lack of clear biomarkers. Digital technologies have great potential to help address some of these deep challenges. However, as researchers, technology developers, disease-focused groups, and others forge forward to take advantage of these tremendous opportunities, it is important to avoid hype and overpromising, and to ensure that this work is done rigorously and collaboratively.

Currently, assessment of function for CNS disorders is based mostly on “snapshots” taken during clinical visits. However, even well-validated instruments may miss overall trends due to variation within and across days, are vulnerable to variations among different clinicians, do not capture activities and function in naturalistic settings, and tend to be biased toward more recent experiences. In addition, these types of assessments generally provide little help in predicting problems during early stages of a disorder, or in helping people and their clinicians detect upcoming periods of relapse in conditions such as schizophrenia.

Digital technologies have the potential to enable reliable, high quality, continuous data collection from large patient populations in their naturalistic settings. In turn, this could help improve our currently limited understanding of natural disease course, patients’ own experiences of the illness, symptom manifestation, and individualized treatment response. Particular opportunities are associated with identifying people in the prodromal period or earlier and in finding early warning signals—such as an impending switch to mania or suicidality—that could enable immediate action. Digital tools could help advance research and clinical care in many other ways as well, including serving as the platform for treatment delivery; Box 1 provides an overview. Digital technologies are being investigated with respect to many different health conditions, but they may be particularly beneficial for addressing CNS disorders. In contrast to many other diseases, it is generally not possible to take brain tissue samples for analysis and there are no blood tests for most CNS disorders, so detection and measurement via digital technology address a fundamental lacuna in this space. In addition, these are multidimensional disorders, often involving circuits and complex behaviors, and digital technologies can provide measurements and potential interventions in multiple domains, including sleep, cognition, social interaction, and others. Finally, interacting with smartphones and social media requires executive function and cognitive control, and therefore provides natural settings for measuring and intervening on brain-based disorders. For example, one can use natural language processing to assess email, text message, and social media content for word usage, grammatical structure, and narrative coherence. In contrast to most physical conditions, certain interventions that are effective for some CNS disorders, such as cognitive behavioral therapy, may be particularly well-suited for delivery via digital technology.

In June 2018, the National Academies of Sciences, Engineering, and Medicine’s Forum on Neuroscience and Nervous System Disorders hosted a workshop that brought together a wide range of experts and stakeholders who are working to examine and take advantage of the promise of digital technologies. Participants came from different sectors (academia, government funders and regulators, industry, and nonprofit) and different disciplines (including neurology, psychiatry, mental health intervention research, biostatistics, computer science, engineering, and law), and they focused on different conditions (neurodegenerative, neuropsychiatric, neurodevelopmental, and substance use) and different technologies and platforms (smartphones, wearables, social media, and environmental sensors). The workshop was an opportunity to take stock of the landscape and work together to discuss how to address both scientific problems and practical challenges, so that the potential of digital technologies could be translated into meaningful contributions toward the health of individuals and society.

Workshop presentations and discussions focused on four key challenges in this domain: transforming data into insight; navigating regulatory pathways in a rapidly-evolving field; designing user-centered tools; and building partnerships in a complex ecosystem. This article provides a high-level view of the many issues, opportunities, and challenges discussed by individual workshop participants. Additional details and attributions of comments to specific participants are available in *Harnessing Mobile Devices for Nervous System Disorders—Proceedings of a Workshop*.^2^

Box 1 The promise of digital tools in research and clinical care *Research: To more deeply understand health and disease and to speed the development of novel therapies*

- Phenotyping diseases in new ways: new techniques & new phenomena
- Documenting disease status (especially early manifestation) and progression
- Serving as endpoints of symptomatic or disease response in interventional trials
- Bringing more relevant, “real-world” evidence of treatment effects, both beneficial and adverse
- Enhancing the efficiency of how studies are conducted and their statistical power
- Improving access to relevant populations
- Increasing diversity of participation in clinical studies
- Enabling the conduct of virtual studies

*Clinical care: To improve the efficiency and efficacy of disease management*

- Providing earlier and more definitive diagnoses
- Delivering more useful clinical phenotyping for prognostication
- Enabling greater precision and more personalization of treatment
- Providing the treatment itself (e.g., providing cognitive behavioral therapy)
- Monitoring response to treatment and disease trajectory to optimize outcomes
- Motivating and engaging patients in their care
- Improving access to expert care
- Facilitating population health management

Source: Adapted from William Marks’ presentation on June 5, 2018 at the Forum on Neuroscience and Nervous System Disorders’ workshop.

## Transforming data into insight

While the promise and potential of digital technologies is relatively intuitive, making digital data useful for research and clinical purposes is significantly more difficult than it might seem at first glance.

To select a device—or multiple devices used together—investigators need to determine which data will be most useful for addressing each intended purpose, which device(s) will enable collection of this data, and how, where, and from whom to collect the data. While multi-domain sensors that capture continuous high-grade data may be most desirable from a research standpoint, many other considerations and potential trade-offs are involved in these decisions, such as those related to battery life, storage of very large data sets, and availability of the technology. To address these issues, it may be beneficial to consider intermittent sampling and to assess whether raw data truly are required to address the question at hand or questions that may emerge later. Incorporating users’ preferences and reducing burden is also critical; several workshop participants noted that passive recording would be the ideal in many cases. Finally, it is important to consider long-term sustainability and business models that support different devices; a “perfect” custom-built device that is no longer available will not benefit patients.

Data must also be validated to be useful for research or clinical practice. Initially, this will require checking that the sensor is actually capturing the data or measurement in question, confirming the accuracy of the measurement, and finally using that data to identify the activity or behavior of interest. For neurological disorders for which “gold standard” measures exist—such as imaging findings, clinical exams, or molecular endophenotypes—the next level of validation would examine how the digital signals, whether taken individually or aggregated, correlate with these existing validated and widely accepted measures. One workshop participant noted, however, that many neuropsychiatric disorders lack such established measures; in these cases, it may be more relevant to look at how digital measures relate to disordered behavior or diagnosable illness, or to consider how they may help select interventions or make decisions regarding incremental care.

Free-form digital data acquired across different contexts and environments are high-dimensional and usually extremely noisy, with substantial variability and unexpected patterns of messiness. Some workshop participants noted that although progress has been made, many difficult challenges remain, such as developing statistical methods to impute missing data and developing robust inferential tools to manage the high variability of data. Integrating data from multiple sources will require creating data standards and establishing platforms that enable data interoperability. Some promising efforts are underway to address these challenges. Throughout the process, it will be important to consider the reproducibility issues that have been raised in the context of machine learning and artificial intelligence (AI) more broadly.^3^ And, finally, significant work still needs to be done in developing approaches to representing complex multidimensional data—for example, data from sensors, clinical assessments, and other outcome measures—in a format from which insights can be gleaned.

## Navigating regulatory pathways in a rapidly-evolving field

Mobile technologies and their applications are evolving faster than drugs and biologics; these rapid changes encompass commercial sensors, proprietary algorithms, and applications that are constantly being changed and updated. And yet several workshop participants emphasized that it remains critically important to ensure accuracy, validity, utility, and value.

From a regulatory perspective, digital health technologies may be defined as medical devices if they are intended for use in the diagnosis, cure, mitigation, treatment, or prevention of disease, or if they are intended to impact the structure or function of the human body through a non-chemical action (For the formal definition of medical devices, see section 201(h) of the Federal Food, Drug, and Cosmetic Act (21 U.S.C. 321).). Medical devices can be classified into one of three categories with different levels of regulatory control based, in part, on the individual risk/benefit profile of the device.^4^ The Food and Drug Administration’s (FDA’s) Center for Devices and Radiological Health has developed guidance on mobile medical applications^5^ and encourages developers to go through the free presubmission process.^6^ This process is designed to help identify and address key issues early in the development process and may be particularly important in this innovative domain. In 2017, the FDA released a Digital Health Innovation Action Plan,^7^ which outlines practical policies and approaches and describes next steps, and has recently announced additional steps to spur innovation in digital health.^8^

Digital technologies can help enable patient-centric drug development through electronic data transmission from patients at home or remote locations and by capturing clinically meaningful measurements continuously in real-life situations. To gain regulatory acceptance for clinical trial use by the FDA’s Center for Drug Evaluation and Research, both interactive and unobtrusive monitoring devices need to be evaluated for reliability, reproducibility, sensitivity, specificity, and clinical meaningfulness. They must also address issues related to data security, privacy, and traceability.^9^ Several organizations are working to advance the appropriate use of mobile technology and digital tools in clinical trial design, including the Clinical Trials Transformation Initiative (https://www.ctti-clinicaltrials.org/) and the Critical Path for Alzheimer’s Disease (https://c-path.org/programs/cpad/).

Complying with an evolving web of laws and regulations presents a substantial challenge, noted one workshop participant. For example, drug and device developers in the United States must comply with the Health Insurance Portability and Accountability Act, the Common Rule, FDA regulations, National Institutes of Health policies, reimbursement policies from Medicare and various state Medicaid programs, and various state health information confidentiality laws. The situation becomes even more complicated when considering international research and markets because these regulations are not harmonized, and because potentially competing policies are emerging globally regarding individuals’ ability to control their own data and biospecimens.

## Designing user-centered tools

Researchers and technology developers envision many opportunities for digital technology to help people with CNS disorders, clinicians, and healthcare organizations, for instance by enabling earlier identification of psychiatric disorders and by helping to optimize care. However, this promise will only be realized if people and organizations actually use the technology.

Consumer attitudes vary with regard to data ownership, access, and privacy. Clinicians may be reluctant to incorporate mobile technologies into their practice for many reasons, including being too busy, already feeling overloaded with electronic health records, concerns about interference with the clinician–patient relationship, lack of experience with new data types, and doubts about patient benefit. Similarly, a workshop participant noted that gaining healthcare organizations’ acceptance of digital technologies will require addressing their own concerns about costs, privacy, and integration with existing systems.

Several workshop participants urged developers to partner with clinicians, patients, and family members, including for selecting outcomes that matter to patients and their families. Technology developers need to understand the attitudes, beliefs, and experiences of the targeted population; develop models that work with clinicians’ workflows, or effectively help individuals self-manage; and test the technologies in real-world settings.

## Moving forward by building partnerships in a complex ecosystem

Several workshop participants emphasized that having a diverse group of stakeholders together from the beginning of the development process was essential. Clinicians, patients, and developers need to work together as partners, as discussed above, to ensure that designs are user-centered, and that security and privacy issues are appropriately handled. Partnerships and consortia can spur innovation and potentially enable the pooling of phenotypic data across many data sets and neurological diseases, a standard practice in genetics, in order to maximize the value of the data. However, many issues need to be worked out in order to move these types of projects forward. One area that generated significant discussion at the workshop is enabling data aggregation from multiple types of sensors and devices. For research purposes, it is important to collect and share raw data, information about the sensor(s) that were used to collect the data, and the context in which data were collected, but this can run counter to the business models of device manufacturers as well as standard practices in academia (although the data sharing movement is changing this culture), and will need to be addressed.

## Conclusion

Digital technologies hold tremendous promise for helping to improve clinical care for individuals affected by CNS disorders, advance our understanding of the science behind CNS disorders, and accelerate therapeutic development. In recent years, the wide availability of technologies such as smartphones, wearables, and social media has provided particular opportunities for observing behavior in naturalistic settings outside of research labs and the clinic, and therefore to date this has naturally been a significant focus of research and efforts to address challenges. Nevertheless, similar concerns related to rigor in data analysis, user-centered design, and the importance of avoiding hype also apply to other rapidly developing digital technologies, such as virtual/augmented reality and AI-driven bots. As the field continues to rapidly evolve, rigorous and collaborative work among all stakeholders—including patients and clinicians—is needed to ensure that these technologies truly will benefit health.
